# Evolutionary game theory and simulations based on doctor and patient medical malpractice

**DOI:** 10.1371/journal.pone.0282434

**Published:** 2023-03-29

**Authors:** Lin Song, Zhenlei Yu, Qiang He

**Affiliations:** 1 Tianjin University of Traditional Chinese Medicine, Tianjin, China; 2 Information Ministry of Library, Qilu University of Technology, Jinan, China; Zhejiang University of Finance and Economics, CHINA

## Abstract

Doctors and patients are the two critical players in medical malpractice. The evolutionary game model of doctors and patients is constructed based on information asymmetry and bounded rationality. The strategy selection problem of the two players in the medical malpractice process was studied. With change in different parameters, the evolutionary equilibrium strategy of the model was demonstrated using Vensim simulation. The results show that the weight, penalty amount, benefits of standardized practices, and patient medical alarm cost of strategies of different doctors are the key factors affecting doctor–patient evolutionary game system. Medical malpractice can be reduced by adjusting the weight of different strategy choices, increasing the penalties for illegal practices, and standardizing medical malpractice costs based on doctors’ standardized practice income. Measures to effectively resolve medical malpractice are proposed by introducing a third-party normative system, establishing a doctor–patient information management system, and improving doctors’ reward and punishment mechanisms.

## Introduction

In recent years, medical malpractice has frequently occurred in China. Examples include the following medical incidents: Hebei (2016), First Hospital of Peking University (2018), Anhui (2019), Civil Aviation General Hospital (2020) and the large-scale medical incident in the Huimin County People’s Hospital of Binzhou City (2017). China’s courts accepted approximately 59,777 medical malpractice cases from 2018 to 2021, with cases increasing annually. These statistics show that medical malpractice in China is increasing. These cases have a substantial negative impact on doctors’ reputation, patients’ trust, and harmony and stability of society. Patients, doctors, hospitals, and the government are dissatisfied with the current status of doctor–patient relationships, and measures must be adopted to manage them. It is important to analyze doctors’ and patients’ strategic choices in medical malpractice and establish a game model of the evolution of medical malpractice they engage in to build a harmonious doctor–patient relationship.

The concept of medical malpractice has not yet been uniformly defined in academia. The term, medical malpractice was first described in 2018 in the medical dispute prevention and handling ordinance in a government document. It was defined as a dispute between a doctor and patient arising from a medical and treatment activity [1]. In 1999, Meadows proposed an alternative dispute resolution tactic except traditional litigation [2]. In 2000, Sieg established a game model to identify trends and settlement strategies when faced with the uncertainty risk in medical malpractice [3]. Since 2000, scholars have started to focus on evolutionary game theory and have used it to study medical malpractice [4, 5]. The information asymmetry of medical malpractice satisfaction [6, 7], medical ethics [8], and other aspects was quantitatively analyzed and evaluated using game models [9] and evolutionary game models [10]. In addition, several mechanisms that may affect cooperative behavior. In 2019, Quan et al. In the stochastic evolution PGG model, the social exclusion mechanism is innovatively introduced. Based on corresponding simulation experiments in carried out in a structured population, the increasing-returns-to-scale effect can greatly reduce the critical values of the amplification factor under which cooperation can emerge [11]. In 2021, Tianyu Ren and Junjun Zheng introduce a tolerance-based expulsion mechanism into the spatial public goods game, reveal that tolerance-based expulsion can significantly foster cooperation and stabilize pure cooperation under negative conditions [12]. In 2022, Li et al. proposed an updating algorithm that show that if mobility is free, there is a window of parameters where synergies with network reciprocity are possible, and where cooperation can be robust and significantly elevated [13]. This paper establishes a 2 × 2 game model considering the doctor–patient relationship. However, there are far fewer doctors than patients. Generally, physician decisions may be influenced by different individuals whose relationships resemble small-world networks. In 2021, Jiang et al. explored contribute to the literature by exploring the role of group size in the collective risk social dilemma and the potential underlying mechanisms through model simulations and human experiments. This simulation results show that reducing the bystander effect by decreasing the group size can solve the collective-risk social dilemma [14]. Some studies have also found that social exclusion as a form of cooperation can significantly promote cooperation through additional cost [15]. Moreover, a fine-tuned interplay between the minority mobility and network reciprocity that are still functioning is most conducive to promoting cooperation, and the limited mobility of minorities could spare public resources in social dilemma situations more effectively than reward and punishment [16]. In recent years, scholars have begun to pay attention to the research of medical malpractice, using path analysis [17], game models [18], and literature reviews [19], and as well as using tolerance-based punishment and cooperation [20], pool expulsion and cooperation [21], prosocial and antisocial exclusion mechanisms [22] to analyze and evaluate factors affecting medical malpractice [23], medical practice income [24], and medical costs [25].

In summary, previous studies have focused on analyzing the game theory between doctors and patients and influencing cooperative behavior mechanisms, which helps reduce medical malpractice. However, few scholars have discussed how doctors’ choice of the combination of two strategies affect patients’ medical behavior. Few studies have used quantitative methods to explore the choice of two different combinations of strategies for physicians, patients’ medical behavior strategies, and how to control the factors affecting medical malpractice to evolve in the direction of established goals.

The main features of this research are as follows:

1. Based on the conditions of bounded rationality, medical malpractice can be interpreted as dynamic games of constant selection and mutation. The evolutionary game method was used to solve the problem of medical malpractice. Therefore, an evolutionary game model of doctors and patients was constructed. The research results present the balanced behavioral choices of doctors and patients, which align with the game situation between doctors and patients in medical malpractice.
2. The choice of stability strategies for doctors and patients in medical malpractice was studied by analyzing the equilibrium point and stability of the evolutionary game model between the players. Second, the model was simulated using Vensim by changing parameters, such as the doctor’s income and patient’s medical challenge. This reflects, intuitively, the evolutionary trend of doctors and patients in the medical malpractice process. Therefore, measures must be taken to alleviate the relationship between doctors and patients to ensure strong operability.

## Methods

### Basic assumptions and model building

Assume that there are two groups of doctors and patients, and one group is randomly selected from the group of doctors each time to match and play with the patients. Doctors and patients have limited rationality, they consider long-term cooperation and learn to constantly change their strategies until equilibrium is reached. The behavior of doctors is divided into: standardized practice and illegal practice, cooperation and conflict. The patient’s behavior is divided into non-medical malpractice and medical malpractice. Doctors’ standardized practices include complying with laws and regulations and ensuring the safety and quality of patients. Collaboration strategies include working with patients and enabling them to cooperate with treatments during the patient care process, ensuring effective communication and good treatment options. The patients’ non-medical behavior includes the behavior of choosing not to engage in medical disputes under the different strategies of the doctors.

In summary, there are two groups of participants in the evolutionary game model: doctors and patients. The doctors’ behaviors were grouped as: standardized practice, illegal practice, and cooperation and conflict. The patients’ behaviors were grouped into non-medical malpractice, and medical malpractice.

Hypothesis 1: The proportion of doctors practicing in a standardized manner is Y_**1**_ (0≤ Y_**1**_≤1), and in an irregular practice is 1-Y_**1**_. The proportion of doctors choosing cooperative strategy is Y_**2**_ (0≤ Y_**2**_≤1), and conflict strategy is 1-Y_**2**_. The proportion of patients who do not raise medical malpractice is X (0≤X≤1), and the proportion of patients with medical malpractice is 1-X.The probability of a doctor choosing an active strategy is Y; the weight of the doctor’s standardized practice and cooperative strategy on the doctor–patient relationship is expressed as S_**1**_ and S_**2**_, respectively.

Hypothesis 2: Some hospitals will engage in illegal practices to maximize benefits but do so at the patient’s expense. Such behaviors include unnecessary medical treatment for patients, prescribing overpriced drugs, and other improper means. These benefits are greater than those under the standardized practice state (recorded as PE_2_>PE_1_). Similarly, doctors choose different strategies in medical malpractice (cooperation or conflict), which will have different effects. In medical services, the cost a conflict between doctors and patients is higher than that of cooperation (denoted as O_2_> O_1_).

Hypothesis 3: Under Article 54 of the Tort Liability Law of the People’s Republic of China, a medical dispute occurs if a patient is damaged in the diagnosis and treatment activities of a medical dispute (this article is recorded as H). If the medical institution and its medical staff are at fault, the medical institution shall bear the liability for damages (this article is recorded as D). Concurrently, the government will oversee and penalize the doctor’s illegal practice under Article 47 of the Regulations on the Prevention and Handling of medical malpractice, which is recorded as M. When a medical malpractice case arises, some patients are dissatisfied with the compensation they receive. If the patient’s expected benefits are inconsistent through the normal channels of rights protection, additional compensation can be sought based on the “medical malpractice” strategy. In the medical malpractice process, the patient’s transportation expenses, lost work expenses, and other costs are recorded (this article is recorded as E).

Hypothesis 4: The patient’s “medical malpractice” behavior will instigate a decline in the doctor’s social reputation, potentially interfering with standard medical processes, and possibly precipitate other losses (this article is recorded as L_1_). For doctors practicing illegally, the patient’s “medical malpractice” will likely expose his/her illegal practice. The doctor’s behavior will be monitored by the government, media, social groups, and others. Therefore, the doctor will suffer greater losses (L_2_, L_2_>L_1_). Furthermore, the “medical malpractice” will not only disrupt the medical order but also negatively impact the harmony and stability of society, doctors’ social standing, and patients’ trust as the incident continues to deteriorate(K).

Based on the above assumptions, a doctor–patient evolutionary game benefit matrix was constructed, as shown in Table 1.

**Table 1:** Payout matrix for doctors and patients in medical malpractice.

Strategies		Payoffs	
P_1_	P_2_	Doctors	Patients
standardized practice, cooperation	non-medical malpractice	PE1-O1-D	D-H
standardized practice, conflict	non-medical malpractice	PE1-O2-D	D-H
illegal practices, cooperation	non-medical malpractice	PE2-O1-D	D-H
illegal practices, conflict	non-medical malpractice	PE2-O2-D	D-H
standardized practice, cooperation	medical malpractice	PE1-O1-KL1-(K+1) D	(K+1) D-KE-H
standardized practice, conflict	medical malpractice	PE1-O2-(K+1) D-KL1	(K+1) D-KE-H
illegal practices, cooperation	medical malpractice	PE2-O1-(K+1) D-M-KL2	(K+1) D-KE-H
illegal practices, conflict	medical malpractice	PE2-O2-(K+1) D-M-KL2	(K+1) D-KE-H

## Evolutionary game analysis of doctors and patients

### Equilibrium point of the evolutionary process

The expected benefits EA_1_ and EA_2_ of the physician’s selection of the normative practice and illegal practice, expected earnings EB_1_ and EB_2_ of the physician’s selection of the cooperation strategy and conflict strategy, and average expected return of the physician (EA)^−^, (EB)^−^ are expressed as:

$$\begin{array}{l} EA_{1}=X(PE_{1}‐O_{1}‐D+PE_{1}‐O_{2}‐D)+(1‐X)[PE_{1}‐O_{1}‐KL_{1}‐(K+1)D+PE_{1}‐O_{2}‐(K+1)D‐KL_{1}]\\ =X[2KL_{1}+2KD)+2PE_{1}‐O_{1}‐O_{2}‐2KL_{1}‐2(K+1)D\\ =2(X‐1)(KL_{1}+KD)‐2D+2PE_{1}‐O_{1}‐O_{2} \end{array}$$ (1)

$$\begin{array}{l} EA_{2}=X(PE_{2}‐O_{1}‐D+PE_{2}‐O_{2}‐D)+(1‐X)[PE_{2}‐O_{1}‐(K+1)D‐M‐KL_{2}+PE_{2}‐O_{2}‐(K+1)D‐KL_{2}‐M]\\ =X(2KL_{2}+2KD+2M)+2PE_{2}‐O_{1}‐O_{2}‐2KL_{2}‐2(K+1)D‐2M\\ =2(X‐1)(KD+M+KL_{2})+2PE_{2}‐2D‐O_{1}‐O_{2} \end{array}$$ (2)

$$\begin{array}{l} {\left(EA\right)}^{-}=Y_{1}EA_{1}+(1‐Y_{1})EA_{2}\\ =(Y_{1}‐1)(2PE_{1}‐2PE_{2}+KL_{1}‐KL_{2}+2D‐M)‐O_{1}‐O_{2}+KD+2PE_{1}+KL_{1} \end{array}$$ (3)

$$\begin{array}{l} EB_{1}=X(PE_{1}‐O_{1}‐D+PE_{2}‐O_{1}‐D)+(1‐X)[PE_{1}‐O_{1}‐KL_{1}‐(K+1)D+PE_{2}‐O_{1}‐(K+1)D‐M‐KL_{2}]\\ =X(2KD+KL_{1}+KL_{2}+M)+PE_{1}+PE_{2}‐2O_{1}‐KL_{1}‐KL_{2}+2(K+1)D‐M\\ =X(X‐1)(2KD+KL_{1}+KL_{2}+M)+PE_{1}+PE_{2}‐2O_{1}‐2D \end{array}$$ (4)

$$\begin{array}{l} EB_{2}=X(PE_{1}‐O_{2}‐D+PE_{2}‐O_{2}‐D)+(1‐X)[PE_{1}‐O_{2}‐(K+1)D‐KL_{1}+PE_{2}‐O_{2}‐(K+1)D‐KL_{2}‐M]\\ =X(2KD+KL_{1}+KL_{2}+M)+PE_{1}+PE_{2}‐2O_{2}‐KL_{1}‐KL_{2}+2(K+1)D‐M\\ =X(X‐1)(2KD+KL_{1}+KL_{2}+M)+PE_{1}+PE_{2}‐2O_{2}‐2D \end{array}$$ (5)

$$\begin{array}{l} {\left(EB\right)}^{-}=Y_{2}EB_{1}+(1‐Y_{2})EB_{2}\\ =2Y_{2}(O_{2}‐O_{1})+X(X‐1)(2KD+KL_{1}+KL_{2}+M)+PE_{1}+PE_{2}‐2O_{2}‐2D \end{array}$$ (6)

According to the Malthusian dynamic equation, the growth rate of the number of physician’s normative practice strategies is equal to EA_1_ minus the average return, the growth rate of the number of physician cooperative strategies is equal to EB_1_ minus the average return, and T is the time, which solves the doctor’s replication dynamic equation:

Let Y represent the probability of the doctor choosing the active strategy, and S_1_ and S_2_ represent the weight of the doctor–patient relationship of the standardized practice and cooperation strategy of the doctor, respectively.


$$Y_{1}=S_{1}Y\;Y_{2}=S_{2}Y$$ (7)

$$Y=\frac{Y_{1}}{S_{1}+S_{2}}+\frac{Y_{2}}{S_{1}+S_{2}}$$ (8)

$$Therefore,\;F(Y)=\frac{F(Y_{1})}{S_{1}+S_{2}}+\frac{F(Y_{2})}{S_{1}+S_{2}}$$ (9)

From this, the replicating dynamic equation for the positive strategy of the medical prescription can be obtained as follows:

$$\begin{array}{l} F(Y)=\mathrm{dY}/\mathrm{dT}=(S_{1}Y‐S_{1}^{2}Y_{2})[2(1‐X)({\mathrm{KL}}_{1}‐{\mathrm{KL}}_{2}‐M)+(2{\mathrm{PE}}_{1}‐2{\mathrm{PE}}_{2}]+S_{2}Y‐S_{2}^{2}Y_{2})(O_{2}‐O_{1})\\ =S_{1}Y[2(1‐X)({\mathrm{KL}}_{1}‐{\mathrm{KL}}_{2}‐M)+2{\mathrm{PE}}_{1}‐2{\mathrm{PE}}_{2}]‐S_{1}^{2}Y_{2}[2(1‐X)({\mathrm{KL}}_{1}‐{\mathrm{KL}}_{2}‐M)+2{\mathrm{PE}}_{1}‐2{\mathrm{PE}}_{2}]+S_{2}Y(O_{2}‐O_{1})‐S_{2}^{2}Y_{2}(O_{2}‐O_{1})/(S_{1}+S_{2}) \end{array}$$

$$\mathrm{Let}\;U=2(1‐X)({\mathrm{KL}}_{1}‐{\mathrm{KL}}_{2}‐M)+2{\mathrm{PE}}_{1}‐2{\mathrm{PE}}_{2},\;\mathrm{and}\;\mathrm{then}$$ (10)

$$F(Y)=dY/dT=Y[S_{1}U+S_{2}(O_{2}‐O_{1})]+Y_{2}[‐S_{1}{}^{2}u‐S_{2}{}^{2}(O_{2}‐O_{1})]$$ (11)

Similarly, the expected benefits of EC_1_ and EC_2_ and the average benefits of patients choosing a non-medical strategy are:

$$EC_{1}=Y_{1}Y_{2}(D‐H)+Y_{1}(1‐Y_{2})(D‐H)+(1‐Y_{1})Y_{2}(D‐H)+(1‐Y_{1})(1‐Y_{2})(D‐H)=D‐H$$ (12)

$$\begin{array}{l} EC_{2}=Y_{1}Y_{2}[(K+1)D‐KE‐H]+Y_{1}(1‐Y_{2})[(K+1)D‐KE‐H)]+(1‐Y_{1})Y_{2}[(K+1)D‐KE‐H)]+(1‐Y_{1})(1‐Y_{2})[(K+1)D‐KE‐H)]\\ =(K+1)D‐KE‐H \end{array}$$ (13)

$$\begin{array}{l} {\left(EC\right)}^{-}=YEC_{1}+(1‐Y)EC_{2}\\ =YK(E‐D)+(K+1)D‐KE‐H \end{array}$$ (14)

The replication dynamic equations for patients are:

F(X)=dx/dt=X(1‐X)K(E‐D) (15)

From the above two equations, a two-dimensional power system (L) can be obtained.


$$\left\{ \begin{array}{l} Y[S_{1}U+S_{2}(O_{2}‐O_{1})]+Y_{2}[‐S_{1}{}^{2}‐S_{2}{}^{2}(O_{2}‐O_{1})]\\ X(1‐X)K(E‐D) \end{array}\right.$$ (16)

To facilitate the analysis of the equilibrium point and stability of the system, we formulate

$$Y^{*}=S_{1}U+S_{2}(O_{2}‐O_{1})/S_{1}{}^{2}U+S_{2}{}^{2}(O_{2}‐O_{1}),0<Y^{*}<1$$ (17)

Proposition 1: The equilibrium point of the system is A(0,0) B(0,Y*)C(1,0)D(1,Y*)

Proof: For a two-dimensional dynamical system (L), with dY/dtT = 0 and dX/dT = 0, (0,0), (0, Y*), (1,0), (1, Y*) are the equilibrium points of the system. Including (0, Y*) and (1, Y*) into the system (L) can also make dY/dT = 0 and dX/dT = 0, that is, four equilibrium points of the system (L) are obtained.

### Equilibrium point and stability analysis

The stability of the evolutionary equilibrium point can be derived from the local Jacobi stability analysis of dynamical systems [26]. Thus, the stable state of the equilibrium point is determined by calculating the eigenvalues of the Jacobi matrix.


$$J=[\begin{array}{ll} \frac{\partial F(X)}{\partial X} & \frac{\partial F(X)}{\partial Y}\\ \frac{\partial F(Y)}{\partial X} & \frac{\partial F(Y)}{\partial Y} \end{array}]$$ (18A)

$$=[\begin{array}{ll} A_{11} & A_{12}\\ A_{21} & A_{22} \end{array}]$$ (18B)

Where A_11_, A_12_, A_21_ and A_22_ expressed as follows:

$$A_{11}=\frac{\partial F(X)}{\partial X}=(1‐2X)K(E‐D)$$ (19)

$$A_{12}=\frac{\partial F(X)}{\partial Y}=x(1‐X)K(E‐D)$$ (20)

$$A_{21}=\frac{\partial F(Y)}{\partial X}=0$$ (21)

$$A_{22}=\frac{\partial F(Y)}{\partial Y}=S_{1}U+S_{2}(O_{2}‐O_{1})+2Y[‐S_{1}{}^{2}U‐S_{2}{}^{2}(O_{2}‐O_{1})]$$ (22)

If both of the following conditions are met, the equilibrium point of the duplication dynamic equation is the evolutionary stabilization strategy (ESS).


$$1.tr\;J=A_{11}+A_{22}<0(trace\;conditions)$$ (23)

$$2.det\;J=A_{11}A_{22}‐A_{12}A_{21}>0(Jacobi\;determinant\;condition)$$ (24)

Therefore, four local equilibrium points can be obtained, i.e., A_11_, A_12,_ A_21_ and A_22_ (as shown in Table 2 below).

**Table 2:** The specific values of A_11_, A_12,_ A_21_ and A_22_ at the local equilibrium point.

Equilibrium point	A_11_	A_12_	A_21_	A_22_
(0, 0)	K(E-D)	0	0	2S_1_(KL_1_-KL_2_-M+PE_1_-PE_2_)+S_2_(O_2_-O_1_)
(0, Y*)	K(E-D)	0	0	-2S_1_(KL_1_-KL_2_-M+PE_1_-PE_2_)-S_2_(O_2_-O_1_)
(1, 0)	-K(E-D)	0	0	2S_1_(PE_1_-PE_2_)
(1, Y*)	-K(E-D)	0	0	-2S_1_(PE_1_-PE_2_)


$$Thereunto,\;Y^{*}=S_{1}U+S_{2}(O_{2}‐O_{1})/S_{1}{}^{2}U+S_{2}{}^{2}(O_{2}‐O_{1})=2S_{1}[(1‐X)\left(KL_{1}‐KL_{2}‐M)+PE_{1}‐PE_{2}]+S_{2}(O_{2}‐O_{1})/2S_{1}{}^{2}[(1‐X\right)(KL_{1}‐KL_{2}‐M)+PE_{1}‐PE_{2}]+S_{2}{}^{2}(O_{2}‐O_{1}),0<Y^{*}<1$$ (25)

According to the determinant values and trace of the Jacobi matrix, the values of the determinant and trace of the Jacobi matrix J at each equilibrium point can be obtained, and the local stability can be judged.

1. When E<D and S_2_(O_2_-O_1_)/2S_1_+ KL_1_-KL_2_-M <PE_2_-PE_1_<0 or E<D and PE_2_-PE_1_>0, the evolutionary stability strategy (ESS) of the system is (0,0).
2. The ESS of the system is (0, y*) when E>D and PE_2_-PE_1_<S_2_(O_2_-O_1_)/2S_1_+ KL_1_-KL_2_-M, or E<D and PE_2_-PE_1_<S_2_(O_2_-O_1_)/2S_1_+ KL_1_-KL_2_-M.
3. When E>D and S_2_(O_2_-O_1_)/S2_1_+ KL_1_-KL_2_-M <PE_2_-PE_1_<0, or when E>D and PE_2_-PE_1_< S_2_(O_2_-O_1_)/2S_1_+ KL_1_-KL_2_-M, the ESS of the system is (1, y*).
4. When E>D and 0<PE_2_-PE_1_<S_2_(O_2_-O_1_)/2S_1_+ KL_1_-KL_2_-M, or when E>D and PE_2_-PE_1_<S_2_(O_2_-O_1_)/2S_1_+ KL_1_-KL_2_-M, the ESS of the system is (1,0).

Based on the two-dimensional dynamic system in the text, the local stability of the Jacobi matrix J can be judged according to the values of each equilibrium point and determinant of the Jacobi matrix. Therefore, according to the values of the determinant and trace of the Jacobian matrix, the first and second cases are shown in Tables 3 and 4, respectively. The evolutionary stability judgment method of the other cases is consistent and will not be described in detail.

**Table 3:** Stability analysis of equilibrium points in case (1).

Equilibrium point	tr J	det J	Stability
(0, 0)	-	+	ESS
(0, Y*)	unsure	-	Saddle point
(1, 0)	+	+	Unstable points
(1, Y*)	unsure	-	Saddle point

**Table 4:** Stability analysis of equilibrium points in case (2).

Equilibrium point	tr J	det J	Stability
(0, 0)	unsure	-	Saddle point
(0, Y*)	unsure	-	Saddle point
(1, 0)	unsure	-	Saddle point
(1, Y*)	+	+	Unstable points

## Results and discussion

### Evolutionary game model based on system dynamics

**Fig 1:**
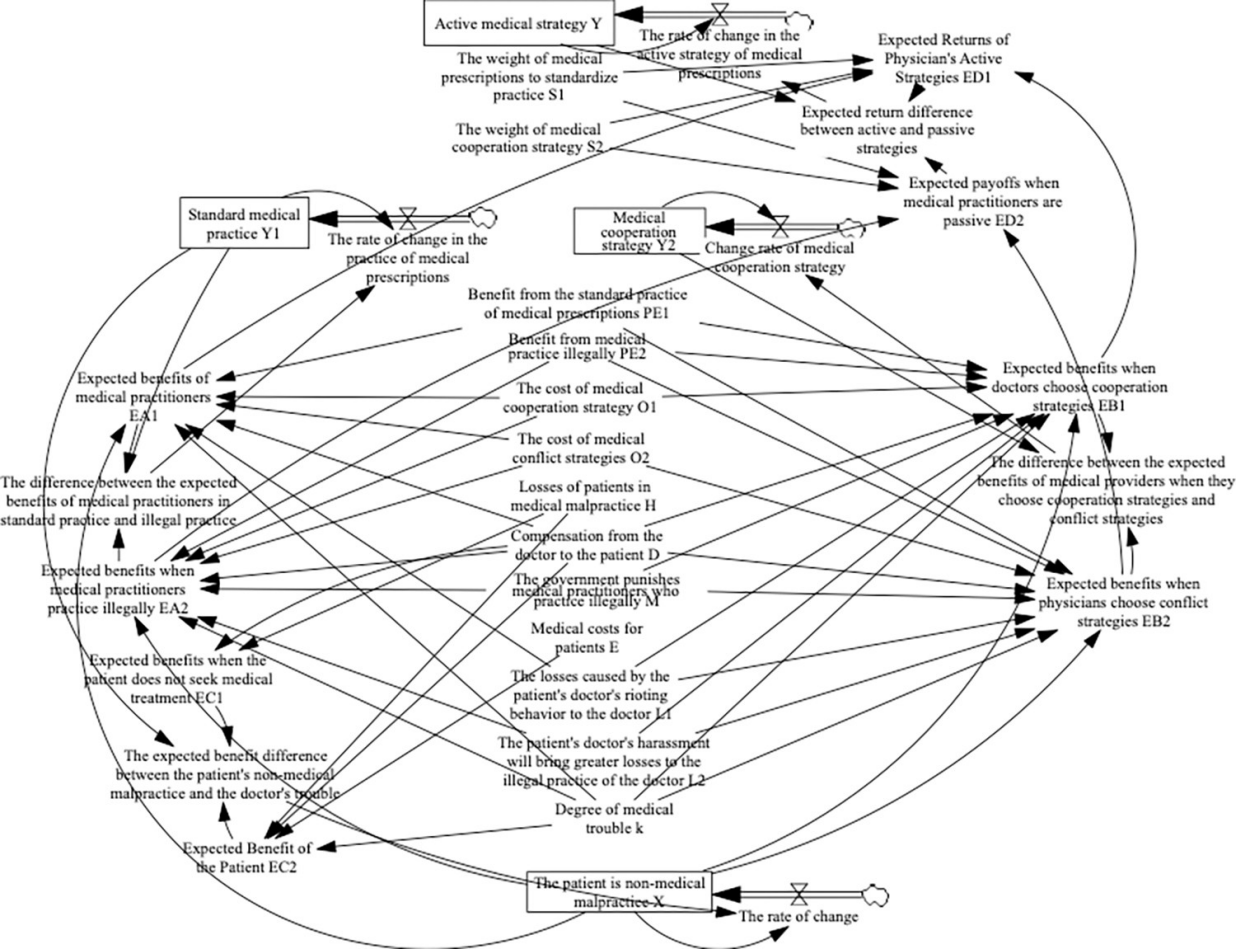
Evolutionary game theory model of doctors and patients in medical malpractice.

The following uses the system dynamics simulation software Vensim PLE to simulate changes in physician and patient strategies. According to the parameters of each game subject and the relationship between parameters, the evolutionary game theory model of doctors and patients is established, (Fig 1).

### Evolutionary simulation research

**Fig 2:**
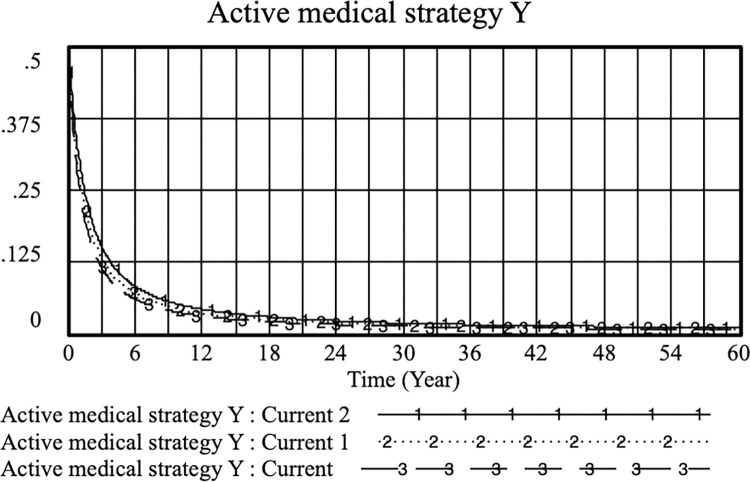
Effect of PE_1_ on the physician positive strategy selection. (current: PE_1_ = 0.4; current 1; PE_1_ = 0.6 and current 2:PE_1_ = 0.8).

**Fig 3:**
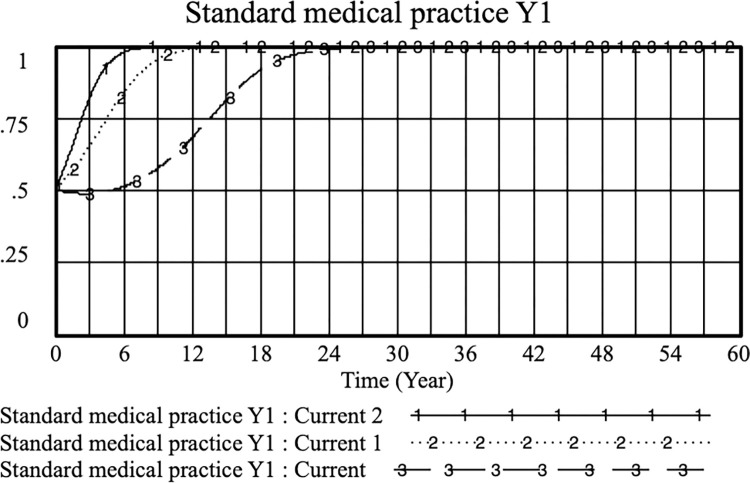
Effect of PE_1_ on the physician standard medical practice strategy selection. (current: PE_1_ = 0.4; current 1; PE_1_ = 0.6 and current 2:PE_1_ = 0.8).

Suppose *K* = 0.4, *S*_*1*_ = 0.5, *S*_*2*_ = 0.5, *PE*_*1*_ = 0.5, *PE*_*2*_ = 0.8, *O*_*1*_ = 0.2, *O*_*2*_ = 0.4, *H* = 0.6, *D* = 0.6, *M* = 0.9, *E* = 0.3, *L*_*1*_ = 0.1, and *L*_*2*_ = 0.3. On this basis, the income (*PE*_*1*_) when doctors regulate their practice is adjusted to observe the change in the probability (Y) of doctors regulating practice with their income (*PE*_*1*_). The simulation results of *PE*_*1*_ of current, current 1 and current 2 were 0.4, 0.6 and 0.8, respectively, as shown in Figs 2 and 3. The higher the income obtained by the doctor’s standardized practice, the higher the probability of choosing to practice in a standardized manner. When the standardized practicing doctor’s income is greater than or equal to that of the illegal practice, the probability of the doctor choosing the standardized practice increases. This shows that effectively controlling and regulating the benefits of standardized practices is conducive to encouraging doctors to provide standardized (legal) strategies as soon as possible.

**Fig 4:**
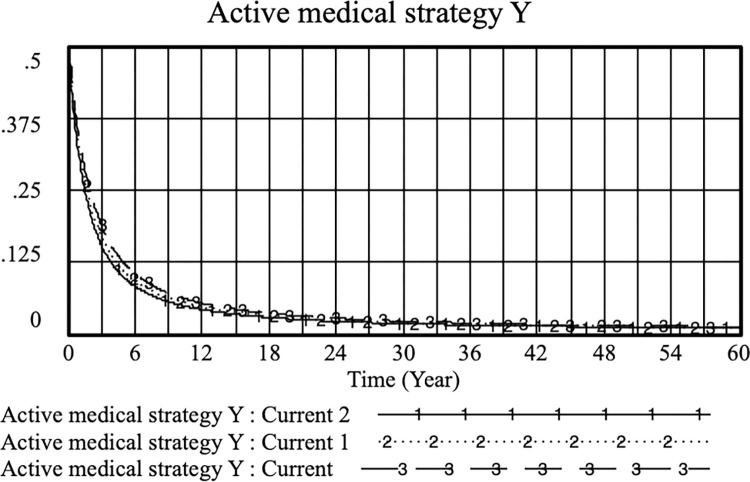
Effects of S_1_ and S_2_ on physicians’ positive strategy selection. (current: S_1_ = 0.1, S_2_ = 0.8; current 1: S_1_ = 0.2, S_2_ = 0.7and current 2:S_1_ = 0.3, S_2_ = 0.6).

**Fig 5:**
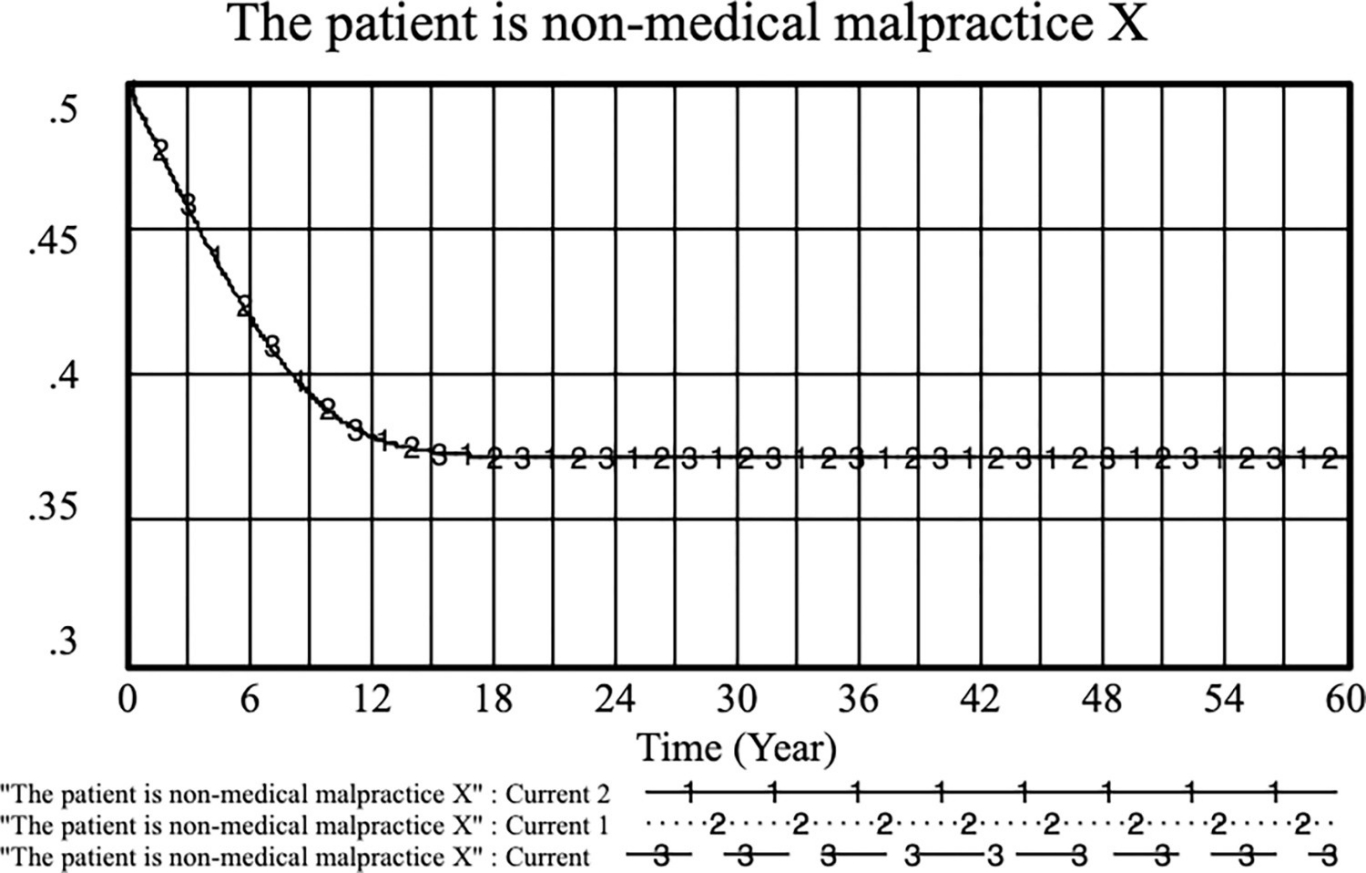
Effects of S_1_ and S_2_ on patients’ strategy selection. (current: S_1_ = 0.1, S_2_ = 0.8; current 1: S_1_ = 0.2, S_2_ = 0.7and current 2:S_1_ = 0.3, S_2_ = 0.6).

The adjusted weights of doctors’ standardized practices after choosing cooperation strategies vary in different cases are as follows: current: *S*_*1*_ = 0.1, *S*_*2*_ = 0.8; current 1: *S*_*1*_ = 0.2, *S*_*2*_ = 0.7and current 2: *S*_*1*_ = 0.3, *S*_*2*_ = 0.6. As shown in Figs 4 and 5, as the doctor’s standardized practice weight *S*_*1*_ declines and the weight of selecting a cooperative strategy *S*_*2*_ increases, the probability of choosing a negative strategy increases. This result indicates that the standardized practice strategy has a high proportion when doctors choose an active strategy. Therefore, by increasing the income of standardized practices between doctors, it is more effective to promote the choice of active strategies by doctors. However, as shown in Fig 4, the changes in S_1_ and S_2_ values do not fluctuate much on the patients’ medical malpractice behavior curve.

**Fig 6:**
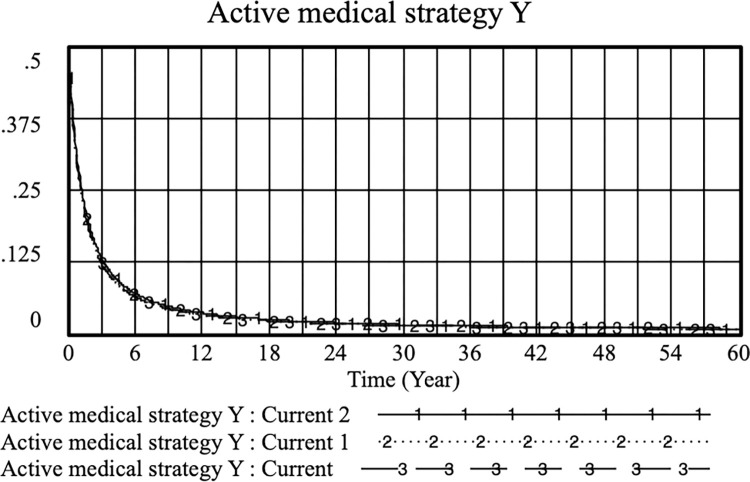
Effects of E and PE_1_ on the choice of active medical strategies for physicians. (current: E = 0.3, PE_1_ = 0.5; current 1: E = 0.4, PE_1_ = 0.5; current 2: E = 0.4, PE_1_ = 0.6).

**Fig 7:**
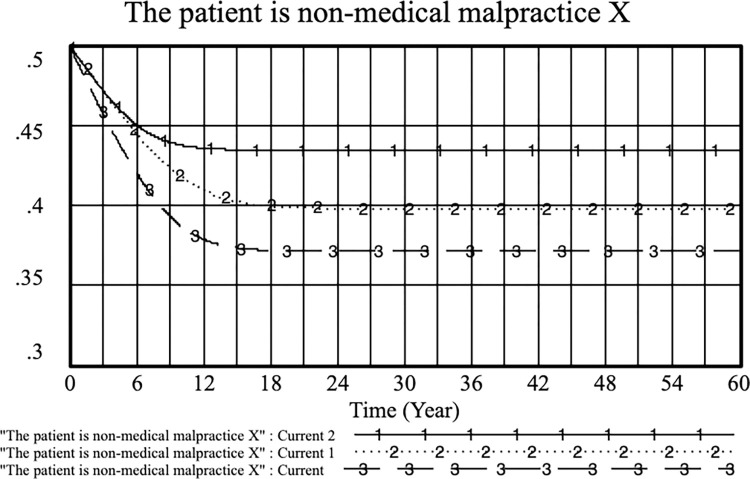
Effects of E and PE_1_ on the choice of non-medical malpractice strategy for patients. (current: E = 0.3, PE_1_ = 0.5; current 1: E = 0.4, PE_1_ = 0.5; current 2: E = 0.4, PE_1_ = 0.6).

By adjusting the patient’s medical alarm cost, the following results were obtained: current: *E* = 0.3, *PE*_*1*_ = 0.5; current 1: *E* = 0.4, *PE*_*1*_ = 0.5 and *PE*_*1*_ when current 2: *E* = 0.4, *PE*_*1*_ = 0.6, improving physician practice. In the case of increasing *E* at the same time, increasing *PE*_*1*_ can improve physicians’ proactive strategies. In this case, controlling the benefits of PE_1_ from the standardized practice of physicians can more effectively increase the probability of patients not filing medical malpractice cases than controlling the cost of medical confusion for patients. As shown in Figs 6 and 7, when the income of doctors to standardize practice (*PE*_*1*_) is between 0.6 and 0.8, patients are more effective in reducing medical malpractice behavior.

## Conclusion

The evolutionary game method is applied to medical malpractice under information asymmetry and limited rationality conditions. The evolutionary game model of doctors and patients was thus established, and a simulation analysis was conducted by determining their respective replication dynamic equations and evolutionary stability strategies. We concluded that the weight of doctors’ standardized practice and cooperation strategies, benefits of doctors’ standardized practice, and patients’ medical noise costs are the key factors affecting the players’ evolutionary game behaviors. Based on the above research conclusions, the measures proposed to resolve medical malpractice from the doctors’ perspective have strong relevance, and the following actions are necessary:

First, third-party supervision and management systems should be improved. In medical malpractice, social media adheres to the principle of objective and fair reporting to assist government management departments in overseeing offending doctors. The government formulates methods and systems for recognizing doctors and patients and raises the threshold for violations. This is done by comparing the situation involving the doctors’ instruments and equipment, staffing, quality, ability, and capital investment. Ensuring that doctors and patients receive the appropriate levels of respect and service, parallel to government-assessed hospital management, will help standardize conducting doctors’ duties.

Second, a doctor–patient information management system should be established. Currently, there is no separate medical malpractice data on the Chinese Central Government’s Official Web Portal. Therefore, to realize the real-time control of medical malpractice by the government and public, a network information platform should be used to build a medical dispute management information system. This platform can enable the timely publication of medical malpractice cases as they occur, effectively manage the medical malpractice database, and reduce government supervision and penalization costs.

Third, doctors’ reward and punishment mechanisms should be improved. Model studies have shown that when a doctor’s gain is substantial, they conduct their duties although the patient is uncooperative. Therefore, a clear penalization system should be established for non-compliant doctors, reducing their number of patients and payment of substantial fines. At the same time, an incentive system should be established for doctors. Bonuses, welfare subsidies, and public awards could be provided to doctors who conscientiously conduct their duties, increasing their trust and credibility over their lifetime and work enthusiasm. The evolutionary game model of doctors and patients established in this study explored the strategy selection problems of two strategies in the process of medical malpractice. The game theory between doctors and patients in medical malpractice also needs to consider government regulations and medical insurance policy, that is, the strategic choice of multi-party stakeholders such as pharmaceutical companies. Thus, it is impossible to analyze various situations in a model. At the same time, this study did not consider that there are plenty of mechanisms that may affect cooperative behaviors, such as network reciprocity, and expulsion. Our next step will be to incorporate it into the model to discuss its impact on medical malpractice. This study provides a basis for further research direction to explore the problem of effective management of medical malpractice by multiple parties.

## Supporting information

S1 Video The effect of PE_1_ on physician positive strategy and standard medical practice strategy selection in evolutionary game theory model of doctors and patients in medical malpractice. (MOV)

S2 Video Effects of S_1_ and S_2_ on physicians’ positive and patients’ strategy selection. (MOV)

S3 Video Effects of E and PE_1_ on the choice of active medical strategies for physicians and non-medical malpractice strategy for patients. (MOV)

S1 Data (ZIP)
