# Supplementary figures and images for: Evolutionary game theory and simulations based on doctor and patient medical malpractice
Source: PLoS One. 2023 Mar 29;18(3):e0282434. doi: 10.1371/journal.pone.0282434 (PMC10057828; doi:10.1371/journal.pone.0282434)

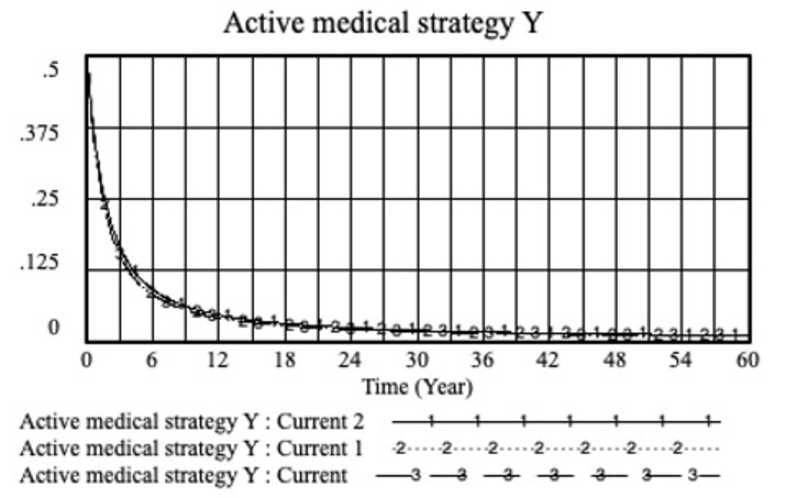

Supplement: S1 Data — (ZIP) [file pone.0282434.s004.zip › raw data/figers/fig 6.tif]

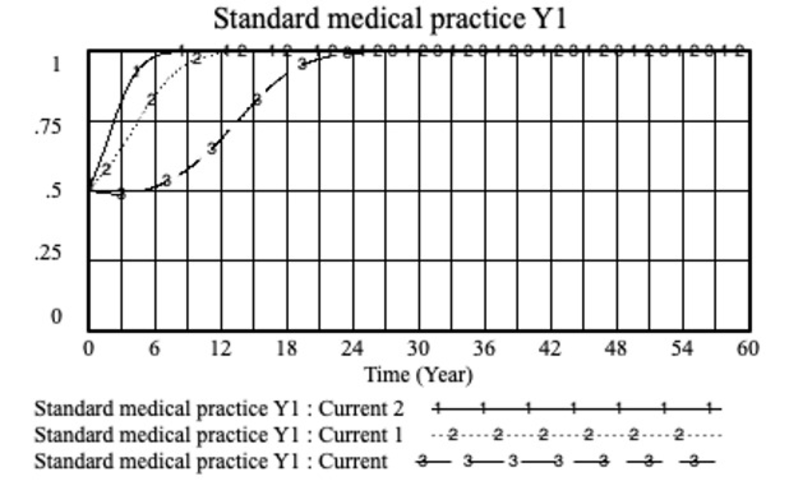

Supplement: S1 Data — (ZIP) [file pone.0282434.s004.zip › raw data/figers/fig3 .tif]

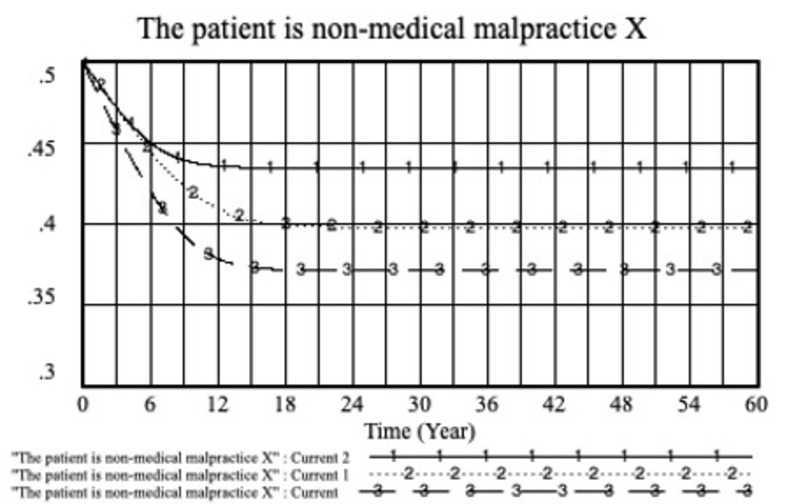

Supplement: S1 Data — (ZIP) [file pone.0282434.s004.zip › raw data/figers/fig7.tif]

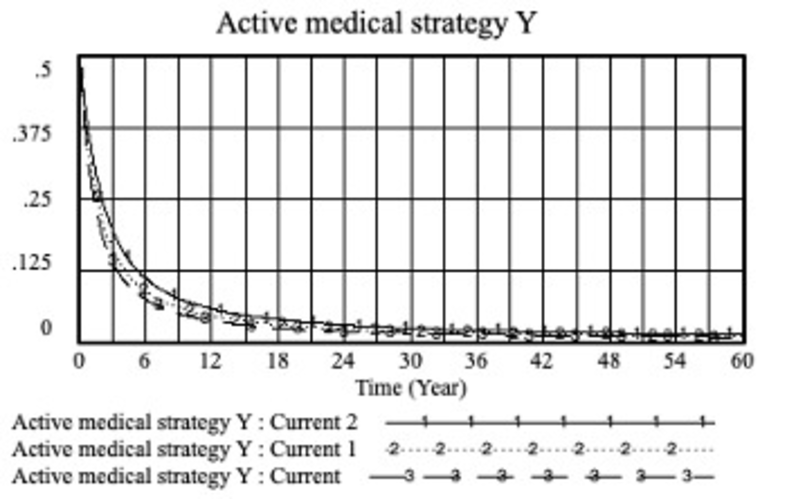

Supplement: S1 Data — (ZIP) [file pone.0282434.s004.zip › raw data/figers/fig 2 .tif]

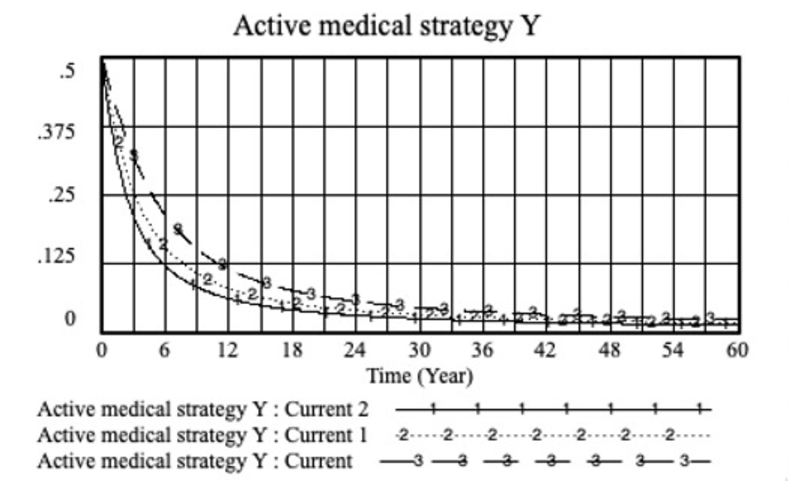

Supplement: S1 Data — (ZIP) [file pone.0282434.s004.zip › raw data/figers/fig 4.tif]

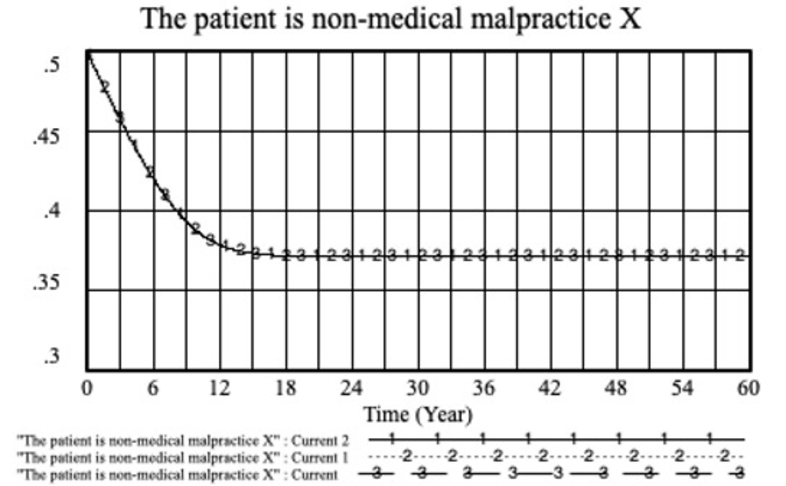

Supplement: S1 Data — (ZIP) [file pone.0282434.s004.zip › raw data/figers/fig 5.tif]

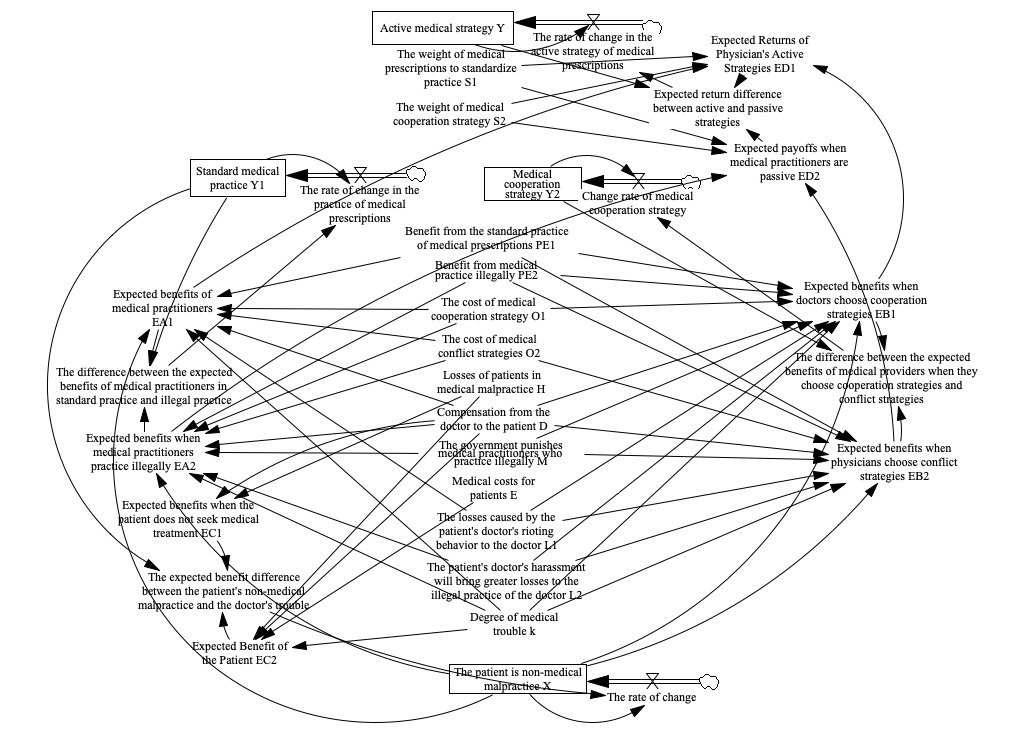

Supplement: S1 Data — (ZIP) [file pone.0282434.s004.zip › raw data/figers/fig 1 .tif]
